# Effect of transmission intensity on hotspots and micro-epidemiology of malaria in sub-Saharan Africa

**DOI:** 10.1186/s12916-017-0887-4

**Published:** 2017-06-30

**Authors:** Polycarp Mogeni, Irene Omedo, Christopher Nyundo, Alice Kamau, Abdisalan Noor, Philip Bejon, on behalf of The Hotspot Group Authors

**Affiliations:** 1 KEMRI-Wellcome Trust Research Programme, CGMR-Coast, Kilifi, Kenya; 2 Centre for Tropical Medicine and Global Health, Nuffield Department of Clinical Medicine, University of Oxford, CCVTM, Oxford, UK; 3 Spatial Health Metrics Group, Kenya Medical Research Institute/Wellcome Trust Research Programme, Nairobi, Kenya

**Keywords:** Malaria, Micro-epidemiology, Hotspots, Sub-Saharan Africa, Stability of hotspots, *D* function, Moran’s *I*, Symptomatic malaria, Asymptomatic parasitaemia, Age, Meta-analysis

## Abstract

**Background:**

Malaria transmission intensity is heterogeneous, complicating the implementation of malaria control interventions. We provide a description of the spatial micro-epidemiology of symptomatic malaria and asymptomatic parasitaemia in multiple sites.

**Methods:**

We assembled data from 19 studies conducted between 1996 and 2015 in seven countries of sub-Saharan Africa with homestead-level geospatial data. Data from each site were used to quantify spatial autocorrelation and examine the temporal stability of hotspots. Parameters from these analyses were examined to identify trends over varying transmission intensity.

**Results:**

Significant hotspots of malaria transmission were observed in most years and sites. The risk ratios of malaria within hotspots were highest at low malaria positive fractions (MPFs) and decreased with increasing MPF (*p* < 0.001). However, statistical significance of hotspots was lowest at extremely low and extremely high MPFs, with a peak in statistical significance at an MPF of ~0.3. In four sites with longitudinal data we noted temporal instability and variable negative correlations between MPF and average age of symptomatic malaria across all sites, suggesting varying degrees of temporal stability.

**Conclusions:**

We observed geographical micro-variation in malaria transmission at sites with a variety of transmission intensities across sub-Saharan Africa. Hotspots are marked at lower transmission intensity, but it becomes difficult to show statistical significance when cases are sparse at very low transmission intensity. Given the predictability with which hotspots occur as transmission intensity falls, malaria control programmes should have a low threshold for responding to apparent clustering of cases.

**Electronic supplementary material:**

The online version of this article (doi:10.1186/s12916-017-0887-4) contains supplementary material, which is available to authorized users.

## Background

Heterogeneity of infectious agents (including *Plasmodium falciparum* malaria parasites) has long been recognized empirically and explored using mathematical models. For many infectious diseases, ~20% of the human population account for ~80% of the infectious burden [1]. Therefore, targeting high-risk populations with effective malaria control measures is likely to be more effective than the same level of untargeted intervention [2]. Furthermore, elimination may not be achieved without some focus on hotspots. Such a strategy requires the accurate identification and a better understanding of the properties of malaria hotspots.

Spatial analyses to describe clustering have been extensively applied in malaria epidemiology [3]. For instance, Kulldorff’s spatial scan statistic and Moran’s *I* statistic have previously been used in malaria epidemiology to demonstrate spatial heterogeneity [4–8]. The scan statistic has been used to identify high-risk areas (’hotspots’) that would potentially benefit from targeted intervention [9], and Moran’s *I* can be used to demonstrate spatial autocorrelation throughout datasets.

Variation in malaria risk has been associated with many factors such as distance to the nearest mosquito breeding grounds (for instance, dams, wet/swampy areas and irrigated farm land) [10, 11], rainfall and temperature [12], altitude [7, 8, 13], proximity to dense vegetation [7, 14], wind direction [15], administration of malaria control interventions (such as insecticide-treated net coverage, indoor residual spraying and anti-malaria drug use) [16, 17], urbanization [18], host genetic factors [19] and human factors including but not limited to social economic status and housing characteristics [7, 20–22]. These factors act at various spatial scales and may explain why some households experience higher risk of malaria while others remain free or experience fewer episodes of the disease.

It has been predicted that hotspots of *P. falciparum* malaria transmission become more marked as transmission intensity declines [2, 23]. Widely used metrics for mapping malaria risk include the prevalence of symptomatic and asymptomatic parasitaemia from cross-sectional surveys [24], fraction of symptomatic malaria cases determined either through the use of case control methods at health facilities [5, 17] or active and passive case detection from cohort studies [4], serological markers and anopheles mosquito abundance conducted using a variety of mosquito collection methods in households [6, 25].

In this study, we describe trends in micro-heterogeneity of malaria transmission using the following empirical data: acute symptomatic malaria (detected through active and/or passive case detection or cross-sectional surveys), and prevalence of parasitaemia detected through cross-sectional surveys. These data are drawn from 19 different study sites across seven sub-Saharan African countries, representing a range of transmission intensities from intense transmission in Burkina Faso [26] to low transmission in The Gambia and the northern part of Kilifi, Kenya [17]. We aimed to (1) examine trends in parameters describing local clustering (or hotspots) and in global measures of spatial autocorrelation of malaria cases at varying transmission intensities, (2) examine temporal stability of hotspots of malaria and (3) investigate the association between micro-variations in mean age of symptomatic malaria (as a proxy for exposure/acquired immunity) and the malaria positive fraction (MPF) across the sites.

## Methods

### Data

**Fig. 1:**
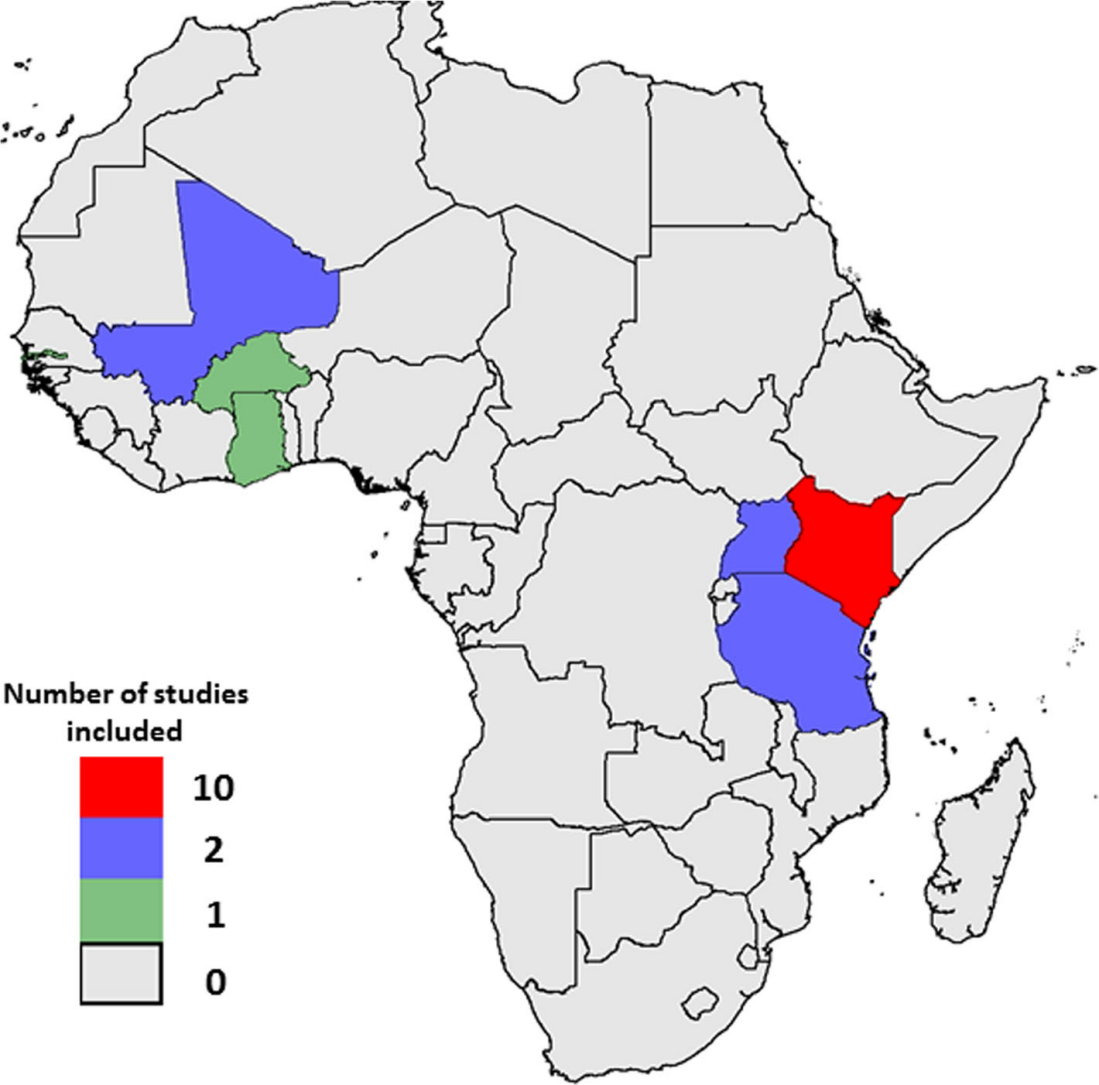
Map of sub-Saharan Africa showing countries and their respective number of studies included in the analysis

Data were assembled from studies conducted in sub-Saharan Africa (Fig. 1, Table 1) with homestead-level geospatial records linked to malaria surveillance at sites with varying transmission intensities. These studies used microscopy for detection of malaria parasites, clinical assessments for presence or absence of fever and reported homestead-level geospatial coordinates. For cluster-randomized or individual-randomized controlled trials, data from intervention and control arms were analysed separately. Datasets were then further divided by year before analysis for spatial clustering. Ethical approval and consent for human participation was granted by relevant authorities of the countries in which the studies were conducted (see references in Table 1). Data were shared with no personal identifiers except geospatial coordinates.

**Table 1:** Study characteristics

Location	Study description	Sample size (*N*)	Malaria case definition (symptomatic malaria)	MPF (*n*/*N*)	Asymptomatic parasitaemia assessed?	Parasite prevalence (%)
Kilifi Kenya [5]	Ngerenya Dispensary Surveillance; monitoring was conducted from 1 April 2014 to 31 December 2015	1998	Any presentation with parasitaemia	0.048	No	Not applicable
Kilifi Kenya [4]	Junju cohort, monitored between 1 January 2005 and 31 December 2015	4534	Temperature >37.5 °C and parasitaemia >2500/μL	0.376	No	Not applicable
Kilifi Kenya [4]	Ngerenya cohort, monitored between 1 January 2003 and 31 December 2015	3659	Temperature >37.5 °C and parasitaemia >2500/μL	0.043	No	Not applicable
Kilifi Kenya [25]	Ganze cross-sectional surveys of asymptomatic parasitaemia and a study cohort monitored for clinical episodes in 2012 and 2013	2532 1518^a^	Temperature >37.5 °C and parasitaemia >2500/μL	0.053	Yes	1.25
Kilifi Kenya [5]	Pingilikani Dispensary Surveillance; monitoring was conducted from 1 January 2009 to 31 December 2014. Each year’s data were analysed separately to capture temporal trend in transmission intensity	22,595	Temperature >37.5 °C and parasitaemia >2500/μL	0.243	No	Not applicable
Kilifi Kenya [17]	Kilifi County Hospital Surveillance; monitoring conducted from 1 January 2009 to 31 December 2014. Each year’s data were analysed separately to capture temporal trends in transmission intensity	8707	Any slide positive test result among acute admissions	0.171	No	Not applicable
Kilifi, Kenya [37]	Junju cross-sectional bleeds between 2011 and 2015, each year’s data were analysed separately to capture temporal trends in transmission intensity	1925	Not applicable	–	Yes	16.05
Nandi, western Kenya [8]	10-week active case surveillance study undertaken in three schools in Nandi District, Western Kenya during a malaria outbreak May to July 2002	520	Temperature >37.5 °C and parasitaemia >2500/μL	0.242	No	Not applicable
Western Kenya [7]	Hospital surveillance study conducted between 2001 and 2004	599	Temperature >37.5 °C and parasitaemia >2500/μL	0.084	No	Not applicable
Asembo, Western Kenya [41]	In late 1996, villages in Asembo were randomized into intervention and control villages. Cross-sectional surveys were conducted between 1996 and 2001. Data from symptomatic and asymptomatic individuals were analysed separately and by year of enrolment.	3614 3047^a^	Measured axillary temperature >37.5 °C and parasitaemia >2500/μL	0.659	Yes	61.9
Rural Afigya-Sekyere, Ghana [14]	Cohort of infants monitored by monthly active case detection and passive case detection. Enrolled at 3 months (±4 weeks) of age between January 2003 and September 2005. Treatment and placebo arms were analysed separately	2721	Temperature >37.5 °C and parasitaemia >500/μL	0.413	No	Not applicable
Mulanda, eastern Uganda [42]	Cross-sectional study conducted in four contiguous villages in Mulanda, sub-county in Tororo, eastern Uganda between July and December 2008.	985	Not applicable	–	Yes	53.7
Uganda [33]	Cohort study of three Uganda sub-counties (Nagongera, Walukuba and Kihihi) between 2011 and 2014	3239	Temperature >37.5 °C and parasitaemia >2500/μL	0.331	No	Not applicable
The Gambia [43]	Cohort study of four Gambian villages (Keneba, Manduar, Jali and Kantong Kunda) between 2009 and 2012	3117	Temperature >37.5 °C and parasitaemia >2500/μL	0.024	No	Not applicable
Mali [44]	Cross-sectional surveys were conducted during the wet and dry seasons and passive case detection in two villages in Mali was conducted between May (Kolle) or July (Sotuba) and December 2009	1867 1128^a^	Temperature >37.5 °C and parasitaemia >2500/μL	0.424	Yes	15.61
Mali [45]	Longitudinal study conducted between May and December 2006. Analysis was restricted to children aged 2-15 years	695 695^a^	Temperature >37.5 °C and parasitaemia >2500/μL	0.51	Yes	21.75
Tanzania [46]	Cross-sectional survey conducted between August and November 2010 in northern Tanzania. Analysis was restricted to children <15 years	328	Not applicable	–	Yes	52.23
Northern Tanzania [47]	The study was conducted between July 2004 and July 2007. Infants aged 2-4 months randomized to treatment regimens. Treatment and placebo arms were analysed separately	2300	Temperature >37.5 °C and parasitaemia >2500/μL	0.161	No	Not applicable
Saponé district, Burkina Faso [26]	Cluster-randomized study with treatment and control arms. Four cross-sectional surveys were conducted between January 2011 and January 2012: (1) before randomization, (2) at 1 month, (3) at 2 months and (4) at 12 months. Monitoring for symptomatic malaria was conducted passively at local health care facilities during the same study period. Treatment and placebo arms were analysed separately	4045 11,932^a^	Temperature >37.5 °C and parasitaemia >2500/μL	0.707	Yes	31.32

^a^Shows sample size for asymptomatic parasitaemia studies when both symptomatic and asymptomatic datasets were available for analysis

### Malaria case definition

Symptomatic malaria and asymptomatic parasitaemia were classified per the definitions shown in Table 1. The key metrics were *P. falciparum* parasite rate (i.e. the proportion of asymptomatic parasite carriage from community cross-sectional surveys), MPF (defined as the fraction of symptomatic malaria) and mean age of children presenting with symptomatic malaria.

### Statistical methods

Data from each site were used to quantify spatial clustering of malaria (described in detail in the following sections). The various metrics from each site were then pooled to examine systematic variation in metrics of spatial clustering over transmission intensity using site as the unit of analysis. Observations with missing geocoordinates, age and malaria slide results in any of the requested datasets were excluded prior to the analysis. No data imputation was done at any analysis stage.

### Local cluster detection

Hotspots are defined as geographical areas experiencing significantly more malaria cases (or more prevalent asymptomatic parasitaemia) than would be expected by chance. Kulldorff’s spatial scan statistic [27], estimated in SaTScan software, was used to detect hotspots. SaTScan imposes a circular scanning window that moves across geographical space with radius varying from zero to a maximum radius enclosing at most 30% (prespecified by the user) of the population in the sampling frame. For each location and size of the window, the number of observed cases was counted, and expected cases were computed by assuming a uniform distribution of cases across the population. The scan statistic compared the count within each circle and that outside to derive a log likelihood statistic. To test the null hypothesis of complete spatial randomness, SaTScan employs multiple random permutations or Monte Carlo simulations based on the observed cases across the entire set of data locations. The observed log likelihood is then compared with the simulated log likelihoods to determine significance [27]. In this analysis, spatial scan statistics were used to detect local spatial clusters of asymptomatic carriers and/or symptomatic malaria cases using a Bernoulli model, where cases were individuals with malaria and controls were individuals without malaria. We assessed variation in the number of hotspots per study site, the risk ratio (RR) of the most likely (primary) hotspot (i.e. the ratio of the risk of malaria within the hotspot divided by the risk outside the hotspot) and the *p* value of the primary hotspot over transmission intensity.

### Global spatial pattern analysis

We used Ripley’s *K* function to analyse binary data (i.e. cases vs controls) and Moran’s *I* for continuous data (e.g. the average ages of children with clinical malaria).

The *K* function [28] was used to test consistency with or departure from spatial randomness within each site. The spatial point pattern data consisted of locations of homesteads with slide positive cases and slide negative controls. The *K* function is a global measure of the number of observed cases within a set of distances of any given case. To control for heterogeneity in the underlying population density distribution, the difference between the *K* function summarizing the degree of clustering of homesteads with cases and controls was computed. Under the null hypothesis of no spatial dependence, the *K* function for cases (*K*_case_(*d*)) and that for the controls (*K*_cont_(*d*)) are identical through the distance (*d*). A difference in *K* function {(*K*_case_(*d*)) – (*K*_cont_(*d*)) [29], also known as the *D* function, greater than zero suggests spatial clustering. The 95% critical regions of the observed *D* functions for the various spatial scales were constructed using repeated simulations. Edge effects due to points close to the boundary of the *K* function were corrected using Besag’s method. Key parameters of interest from this analysis were the estimate of the *D* function and its significance. A sensitivity analysis was conducted at various predefined distances.

Moran’s *I* tests the null hypothesis that there is no spatial clustering of a metric [3]. The test examines whether values among neighbouring homesteads/locations are spatially auto correlated (clustered), random or dispersed. The Moran’s *I* statistic ranges between –1 and 1. A positive Moran’s *I* indicates a tendency towards spatial clustering, a negative Moran’s *I* indicates a tendency towards regularity (dispersion) while a value of 0 indicates a random distribution of events.

Symptomatic malaria cases and asymptomatic parasitaemia were examined separately. For each dataset, parameters from the local cluster detection and from the *D* function analyses were assessed against the overall transmission intensity, measured by the MPF for datasets on symptomatic malaria or parasite prevalence for datasets on asymptomatic parasitaemia.

The multiple fractional polynomial algorithm was used as previously described [30, 31]. Briefly, a list of fractional polynomial (FP) powers (–2, –1, –0.5, 0, 0.5, 1, 2, 3) were examined for possible inclusion in the model using an algorithm that combines a backward elimination procedure with a search for an FP function that best predicts the outcome variable. The deviance difference test statistic is computed for significance testing to determine the final parsimonious model. The multiple polynomial is retained only where log likelihood ratio testing of the nested model shows a statistically significant improvement over the linear model [32]. We used multiple FPs to assess nonlinear fits of MPF or parasite prevalence on the hotspots parameters (i.e. number of hotspots, RRs and *p* values) in the regression models adjusted for potential confounders (i.e. study design, sample size and overall mean age of study participants included in each study).

### Temporal stability of hotspots analysis

There were few datasets with repeated sampling of overlapping homesteads, and therefore stability of spatial heterogeneity could only be tested in four datasets from western Kenya [24], Ghana [14], Burkina Faso [26] and Uganda [33]. MPFs and/or parasite prevalence were computed by grids (2 × 2 km square) and by year (or time points for cross-sectional surveys). We assessed stability of the spatial heterogeneity by examining correlation between MPFs or parasite prevalence within grids separated in time.

The average age of children with malaria was computed as the geometric mean age of children presenting with symptomatic malaria. The correlation between the average age of children with symptomatic malaria and MPF at predefined square grid sizes (i.e. 1 km^2^, 2 km^2^ and 4 km^2^) was calculated using the Spearman’s rank correlation coefficient. Variable grid sizes were used for sensitivity analysis and were calculated using longitude and latitude coordinates. Pooled correlations for the predefined grid size were estimated in a fixed effect meta-analysis; however, if heterogeneity (*I* ^2^) between studies was large (>50%), a random-effect meta-analysis was conducted. Global spatial autocorrelation for age of symptomatic malaria at homestead level within sites was assessed using Moran’s *I* statistic and the significance determined using the Monte Carlo simulations.

SaTScan was executed from R using the rsatscan package, which allows SaTScan to be executed in the background from R’s command line. The *K* function and the Moran’s *I* statistics were executed in R version 3.3.1, and graphs, meta-analyses, multiple FP procedure and other analyses were conducted in Stata version 12 (StataCorp, College Station, TX, USA).

## Results

### Malaria morbidity in the study sites

We had access to data from 19 studies conducted between 1996 and 2015 in seven countries (Fig. 1). The characteristics of each study population are presented in Table 1 with references to previously published work.

### Hotspots and clustering of malaria cases

**Fig. 2:**
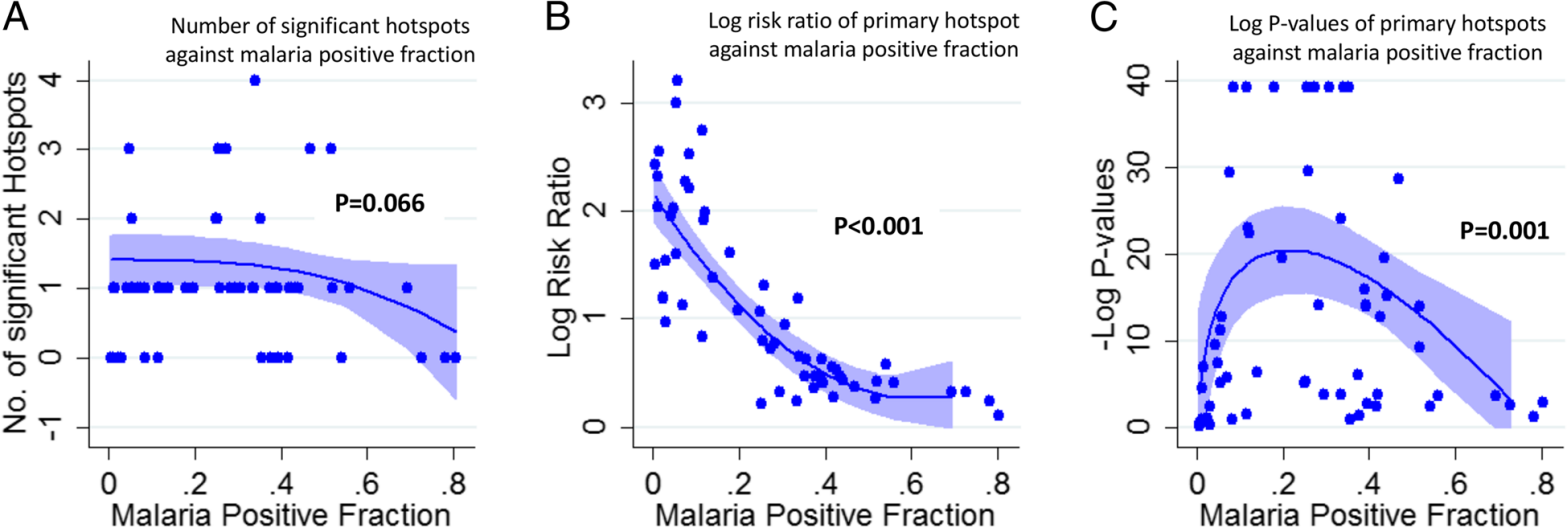
Hotspots of symptomatic parasitaemia. **a** displays a scatter plot of the number of significant hotspots per study area against malaria positive fraction, **b** shows the log risk ratios of malaria within the primary hotspot against the malaria positive fraction and **c** shows the –log (*p* values) of the primary hotspots against malaria positive fraction. The *blue lines* in **a**, **b** and **c** show the fitted multiple fractional polynomial model predictions after adjusting for study design and the overall age of study participants. *Shaded areas* in **a**, **b** and **c** represent 95% confidence intervals (*CIs*)

The median number of significant hotspots for the datasets was 1, and there was no clear trend according to transmission intensity (Fig. 2a). However, the RRs for primary hotspots were highest at low MPFs (Fig. 2b) and decreased with increasing MPF. The statistical significance of hotspots was lower at very low MPFs; it then increased with increasing MPF to a peak at an MPF of ~0.3 and then gradually decreased with increasing MPF after MPF >0.3 (Fig. 2c). Although average age of children in the dataset was significantly associated with the RR and *p* values (Additional file 1: Figure S1), analyses adjusted for average age of children in the dataset (Fig. 2a, b and c) and analyses stratified by study design (i.e. passive vs active case detection) showed a similar trend in variation of RR over transmission intensity (Additional file 2: Figure S2). While there were fewer studies that included data on asymptomatic parasitaemia, a similar trend for RRs with increasing parasite prevalence was observed, but without a clear trend for *p* values (Additional file 3: Figure S3). FP transformations significantly improved model fits (Additional file 4: Table S1).

Using the modelled relationship between *Plasmodium falciparum* parasite rate (PfPR) and *R*_0_ reported by Smith et al. [34], we determined the ratios of *R*_0_ inside to outside the hotspot, and plotted these against PfPR (Additional file 5: Figure S4). The ratio of *R*_0_ inside to *R*_0_ outside rose steeply below a parasite prevalence of 10%, suggesting that the potential to interrupt transmission by targeting hotspots increases below this prevalence.

### Spatial autocorrelation

**Fig. 3:**
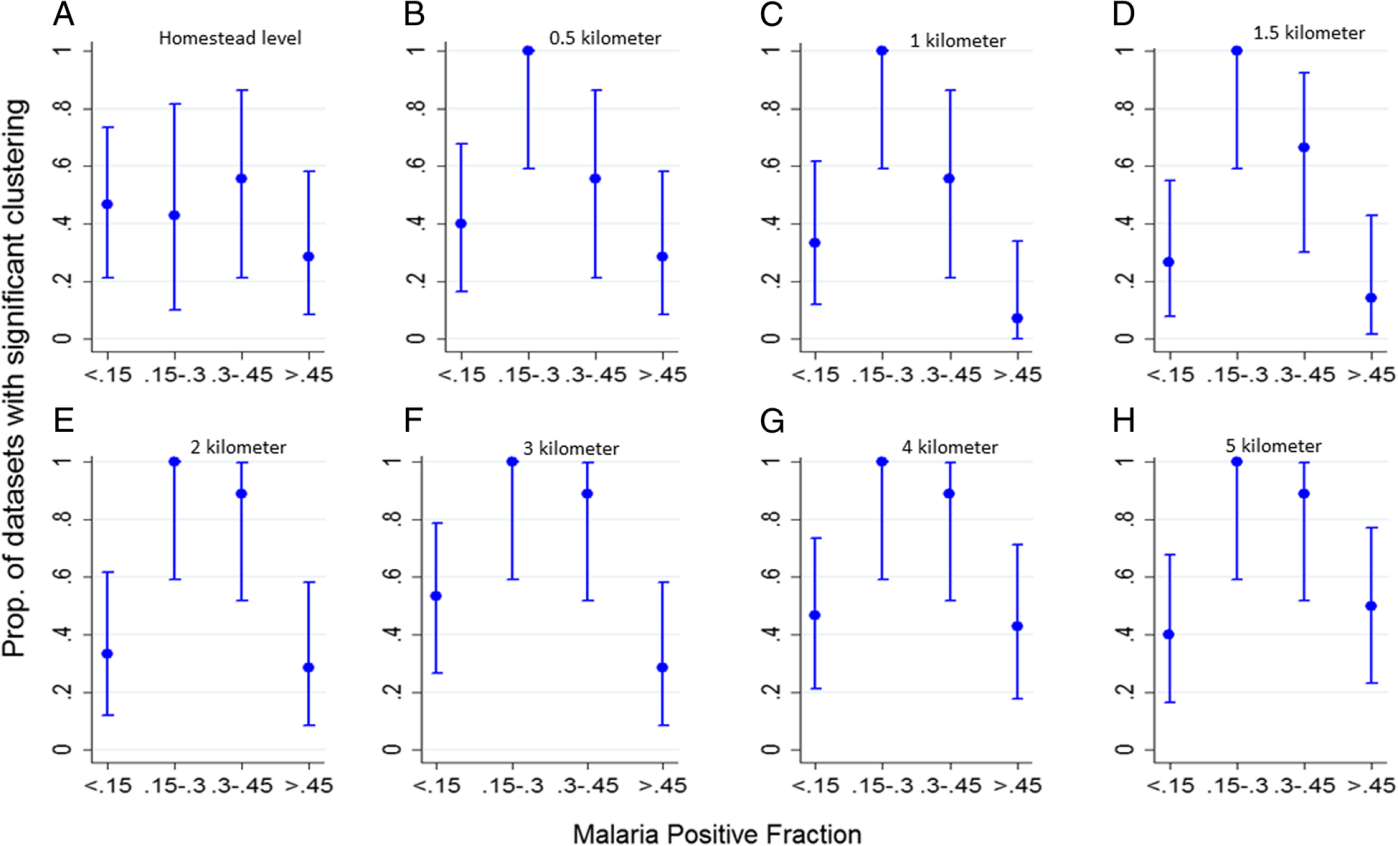
Clustering of malaria transmission. **a**, **b**, **c**, **d**, **e**, **f**, **g** and **h** show the proportion of datasets with significant clustering at homestead level, 0.5, 1, 1.5, 2, 3, 4 and 5 km level respectively, against malaria positive fraction

Ripley’s difference in *K* function (i.e. the *D* function) indicated significant spatial structure in many but not all sites (Fig. 3). As seen with hotspots, the proportion of sites that had significant spatial structure increased from the lowest MPFs to a peak at MPFs of 0.15– 0.45, and then declined at higher MPFs. This trend was consistent at various spatial scales examined (Fig. 3). The magnitude of the *D* function decreased with increasing MPF and was consistent at various spatial scales (Additional file 6: Figure S5).

### Temporal trends

Overall, the spatial distribution of asymptomatic parasitaemia showed modest temporal stability in Asembo and Burkina Faso sites (Table 2). On the other hand, the spatial distribution of febrile malaria was predictive of febrile malaria over 1 and 2 years in Uganda, but not in the other sites.

**Table 2:** Association between distribution of MPF within grids (size = 2 × 2 km^2^) over time interval (Asembo Bay, Kenya [24], sub-counties of Uganda [33] and Afigya-Sekyere Ghana [14]) in years and over consecutive cross-sectional surveys conducted over a span of 1 year (Saponé district, Burkina Faso [26])

Study Site	Interval between cluster (year)	Febrile Malaria		Asymptomatic Parasitaemia	
		Correlation (95%CI)	*P*-value	Correlation (95%CI)	*P*-value
Asembo Bay	1	–0.09 (–0.26 to 0.09)	0.3072	0.23 (0.08 to 0.36)	0.003
	2	0.14 (–0.04 to 0.31)	0.1245	0.16 (0.01 to 0.31)	0.0433
	3	0.16 (–0.08 to 0.38)	0.1873	0.02 (–0.18 to 0.22)	0.8512
	4	0.45 (0.11 to 0.70)	0.0124	0.21 (–0.12 to 0.49)	0.2041
	5	0.06 (–0.32 to 0.43)	0.7726	0.45 (–0.13 to 0.80)	0.1226
Burkina Faso	Interval between clusters (surveys)				
	1	–0.07 (–0.21 to 0.08)	0.3667	0.24 (0.10 to 0.36)	<0.001
	2	0.06 (–0.13 to 0.24)	0.5359	–0.09 (–0.25 to 0.08)	0.293
	3	0.27 (0.01 to 0.50)	0.0457	0.34 (0.11 to 0.53)	0.0043
Uganda	Interval between clusters (years)				
	1	0.39 (0.27 to 0.50)	<0.001	–	–
	2	0.29 (0.13 to 0.44)	0.001	–	–
	3	0.19 (–0.06 to 0.41)	0.1332	–	–
Ghana	Interval between clusters (years)				
	1	0.26 (–0.03 to- 0.51)	0.0756	–	–
	2	0.30 (–0.14 to 0.64)	0.1757	–	–

### Average age of symptomatic malaria episodes and correlations with MPF

**Fig. 4:**
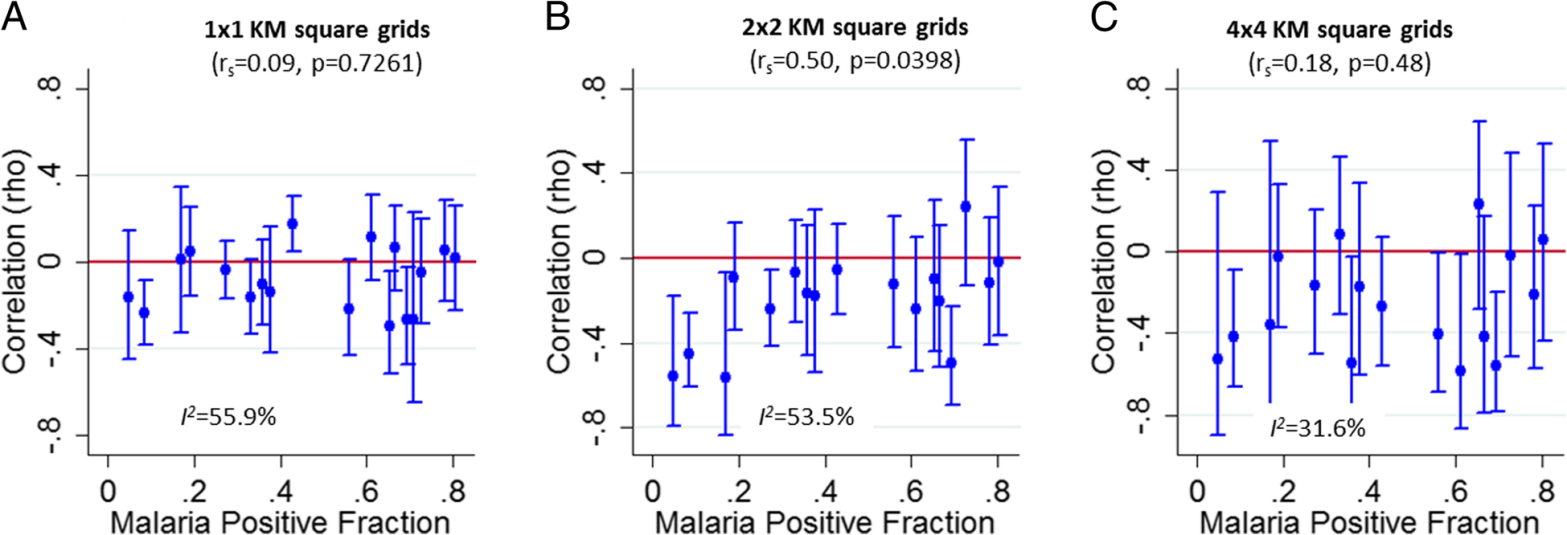
Fine-scale geographical correlation of mean age (months) against malaria positive fraction (*MPF*) for each study dataset plotted against overall study MPFs (as a proxy for transmission intensity). **a**, **b** and **c** show 1 × 1, 2 × 2, and 4 × 4 km^2^ grids respectively. The test of heterogeneity between studies was *I* ^*2*^ = 55.9%, *p* = 0.002; *I* ^*2*^ = 53.5%, *p* = 0.005; and *I* ^*2*^ = 31.6%, *p* = 0.104 respectively

Spearman’s rank correlation coefficients of MPF against average age of symptomatic children with malaria at various spatial scales (i.e. 1 km^2^, 2 km^2^ and 4 km^2^ grids) were negative in most study datasets. This suggests that patches of greater exposure to malaria (i.e. high MPF) were associated with younger children presenting with malaria parasites in their blood and vice versa (Fig. 4). These negative associations tended to be more marked where the average MPF at the site was low, and this trend was significant when the correlations were measured using 2 km^2^ grids (i.e. *p* = 0.04) but not at 1 km^2^ or 4 km^2^ grids (Fig. 4). The pooled correlation between MPF and slide positive age for 1 × 1, 2 × 2 and 4 × 4 kilometer spatial resolution was –0.07 (95% confidence interval (CI) –0.14 to 0.00), –0.21 (95% CI –0.31 to –0.11) and –0.27 (95% CI –0.37 to –0.18) respectively and in the same direction. The test of heterogeneity between studies was *I* ^*2*^ = 55.9%, *p* = 0.002; *I* ^*2*^ = 53.5%, *p* = 0.005; and *I* ^*2*^ = 31.6%, *p* = 0.104 respectively.

Furthermore, we observed significant spatial autocorrelation for the age of symptomatic malaria episodes at most sites (Additional file 7: Figure S6), suggesting that there are focal areas where older individuals tend to be seen with symptomatic malaria, and conversely focal areas where younger individuals tend to be seen with symptomatic malaria.

## Discussion

This study describes fine-scale spatial heterogeneity of *P. falciparum* malaria cases from studies conducted at 19 different study sites experiencing varying transmission intensities in seven sub-Saharan African countries (Fig. 1). The RR of the primary hotspots increased with falling MPF. The strength of evidence (*p* values) increased from low MPFs to moderate MPFs and then declined towards high MPFs. Taking these findings on variation in degree of heterogeneity and on statistical significance of heterogeneity together, we conclude that spatial heterogeneity becomes gradually more marked as transmission intensity falls, albeit with statistical significance becoming weaker at very low transmission intensity because of reduced power resulting from small numbers of malaria cases. It may therefore be appropriate for malaria control programmes to target hotspots at low transmission intensity despite apparently modest statistical significance. The decline in the degree of spatial heterogeneity towards high MPF may be due to either more even distribution of transmission intensity per se, or to saturation in the metric used to quantify malaria exposure (i.e. the MPF).

Similar findings were seen for generalized measures of spatial autocorrelation where the degree of spatial autocorrelation (*D* functions) is shown to increase as MPF falls (Additional file 6: Figure S5) with significance testing showing a peak when MPF is within 0.15–0.45 range (Fig. 3).

Hotspots of stable asymptomatic parasitaemia have previously been described in Kilifi, Kenya [4]. We could quantify the temporal stability of the spatial distribution in four datasets outside Kilifi (where these studies have previously been conducted [14, 24, 26]). The results showed temporal instability; however, with four datasets we were unable to examine trends in stability across sites. An indirect approach to examining temporal stability is to look for evidence of spatial variation in clinical immunity. Micro-variation of malaria transmission is likely to lead to variations in the rate and degree of acquisition of clinical immunity if the micro-variation is sufficiently stable. Children acquire immunity against symptomatic malaria following repeated exposure. At high transmission intensity, children acquire immunity rapidly due to intense exposure when they are young and hence do not present with symptomatic malaria when they are older. On the other hand, at low transmission, children acquire immunity slowly and are more likely to present with symptomatic malaria when they are older [35]. As might therefore be predicted, we observed a negative correlation between MPF and age of symptomatic malaria in keeping with previously reported findings [5, 17], and we took this to imply that immunity is acquired more rapidly with greater exposure to malaria, leading to a lower age of symptomatic malaria episodes. Furthermore, there were positive autocorrelations (i.e. significant values of Moran’s *I*) in the age of children with symptomatic malaria, again suggesting that micro-variation of transmission intensity may have led to variation in the degree of acquisition of clinical immunity.

In the four datasets with longitudinal data, the temporal stability of the distribution of clinical malaria was lower than that seen in previous analyses in Kilifi and the highlands of western Kenya [4, 7]. Furthermore, we identified substantial heterogeneity in the correlations between MPF and age of symptomatic malaria. Taking these findings together, we conclude that temporal stability of hotspots is not a reproducible feature of malaria transmission. We did not identify a strong trend of greater spatial stability at any range of MPF (Fig. 4).

Mathematical models suggest that targeting control interventions on hotspots results in a more marked decline in malaria compared to untargeted interventions with an equal amount of resources [2]. To implement such a strategy requires the accurate identification of hotspots, and our data suggest that hotspots may not be temporally stable and may be more difficult to accurately identify at high transmission. A previous attempt on targeting hotspots of malaria transmission in Rachuonyo — an area of moderate transmission intensity in western Kenya — achieved modest reductions in transmission inside the targeted hotspots but no lasting reductions outside the targeted hotspots in a cluster-randomized control trial [9]. The authors suggested that the limited impact was at least partly explained by challenges in defining the geographical boundaries of transmission hotspots [9]; our findings on temporal instability of hotspots would confirm difficulties in defining hotspots.

Study limitations include the use of data collected using microscopy, which is of limited sensitivity for parasitaemia. Polymerase chain reaction has been shown to be more sensitive for parasitaemia, particularly in low transmission regions [36]. This is unlikely to bias studies based on febrile malaria episodes since symptomatic malaria individuals usually have parasite densities well above the detection threshold. However, sub-microscopic infections among studies of asymptomatic parasitaemia may influence the stability of hotspots. Most studies included applied a threshold parasitaemia to define febrile malaria. The threshold reduces the likelihood that cases of asymptomatic parasitaemia with co-incident fever are non-specifically included in febrile malaria cases [37, 38].

The modifiable areal unit problem may lead to bias when an arbitrary grid size is used to aggregate data. We mitigate this problem by conducting a sensitivity analysis using grids with varying sizes (i.e. 1-km, 2-km and 4-km squares). A further limitation is that detection of febrile malaria is influenced by study design, sample size and targeted age group, which was not standardized between studies. However, we showed similar results even after adjusting for these potential confounders (Fig. 2), and we identified similar results for studies of febrile malaria and of asymptomatic parasitaemia.

Most studies included were conducted in relatively high to moderate transmission settings or in low transmission settings following recent reductions in transmission. Areas that have historically experienced low transmission may not be represented. Furthermore, study sites were grouped in west and east Africa without representation of central and southern Africa.

## Conclusions

We found geographical micro-variation in malaria transmission within sites from across sub-Saharan Africa at a variety of transmission intensities. Micro-variation was greater in low transmission settings, albeit with less statistical power to detect it where cases of malaria are few. The temporal instability of hotspots and the difficulties in defining hotspots (especially in higher transmission settings) will be a challenge to targeted control. However, given the predictability with which hotspots occur as transmission intensity falls, malaria control programmes should have a low threshold (PfPR <10%) for responding to apparent clustering of cases. Many sub-Saharan African countries currently contend with high malaria transmission and, based on recent evidence [9], are unlikely to benefit significantly from targeted control. However, some countries have witnessed substantial declines (such as Zanzibar [39] and Swaziland [40] among others) that warrant the implementation of targeted control to achieve elimination. Our data predict that hotspots will be a marked feature of transmission in such settings.

## Additional files

Additional file 1: Figure S1. Trends in parameters of primary hotspots over mean age of study participants. Panel A shows a scatter plot of log-transformed risk ratios against overall mean age. Panel B shows a scatter plot of log-transformed *p* values against overall mean age. The *green line* presents multiple fractional polynomial fits of age on malaria positive fraction (*MPF*) adjusted for the study design. *Shaded areas* in panels A and B represent 95% CIs. (TIFF 573 kb)

Additional file 2: Figure S2. Summary of malaria hotspots from symptomatic parasitaemia among passive (*blue*) and active (*red*) surveillance studies. Panel A shows a scatter plot of the number of significant hotspots against malaria positive fraction, panel B presents the log risk ratios of malaria within the primary hotspot against the malaria positive fraction and panel C presents the –log *p* values of the primary hotspots against malaria positive fraction. The *blue* and *red lines* in panels A, B and C show the fitted multiple fractional polynomial model predictions for passive and active case detection studies respectively. *Shaded areas* in panels A, B and C represent 95% CIs. (TIFF 821 kb)

Additional file 3: Figure S3. Hotspots of asymptomatic parasitaemia. Panel A displays a scatter plot of the number of significant hotspots in each study dataset against parasite prevalence, panel B presents the log risk ratios of malaria within the primary hotspot against the parasite prevalence and panel C displays the –log (*p* values) of the primary hotspots against parasite prevalence. The *blue lines* in panels A, B and C show the fitted multiple fractional polynomial model predictions. *Shaded areas* in panels A, B and C represent 95% CIs. (TIFF 767 kb)

Additional file 4: Table S1. Comparison between linear and the multiple fractional polynomial model fit. The *p* value shown derives from the log likelihood ratio test for a nested model with a fractional polynomial over the linear fit. (DOCX 12 kb)

Additional file 5: Figure S4. Scatter plot of the ratio of log-transformed *R*_0_ inside to outside the hotspot plotted against parasite prevalence. The *blue line* shows the fitted multiple fractional polynomial model predictions, and the *shaded area* represents 95% CIs. (TIF 1338 kb)

Additional file 6: Figure S5. Difference in *K* functions for cases and controls (*D* function) against malaria positive fraction. Panels A, B, C, D, E, F, G and H show the *D* function at homestead level, 0.5, 1, 1.5, 2, 3, 4 and 5 km distances for each dataset. The *blue dots* represent symptomatic parasitaemia datasets, while *red dots* represent asymptomatic parasitaemia datasets. (TIF 216 kb)

Additional file 7: Figure S6. Homestead level spatial autocorrelation of age in months for symptomatic individuals for the various studies. *Red dots* show significant autocorrelation, while *blue dots* show non-significant spatial autocorrelation. (TIF 90 kb)

## Abbreviations

FP: Fractional polynomial
km: Kilometer
MPF: Malaria positive fraction
PfPR: *Plasmodium falciparum* parasite rate
RR: Risk ratio
