# Supplementary figures and images for: Effect of transmission intensity on hotspots and micro-epidemiology of malaria in sub-Saharan Africa
Source: BMC Med. 2017 Jun 30;15:121. doi: 10.1186/s12916-017-0887-4 (PMC5492887; doi:10.1186/s12916-017-0887-4)

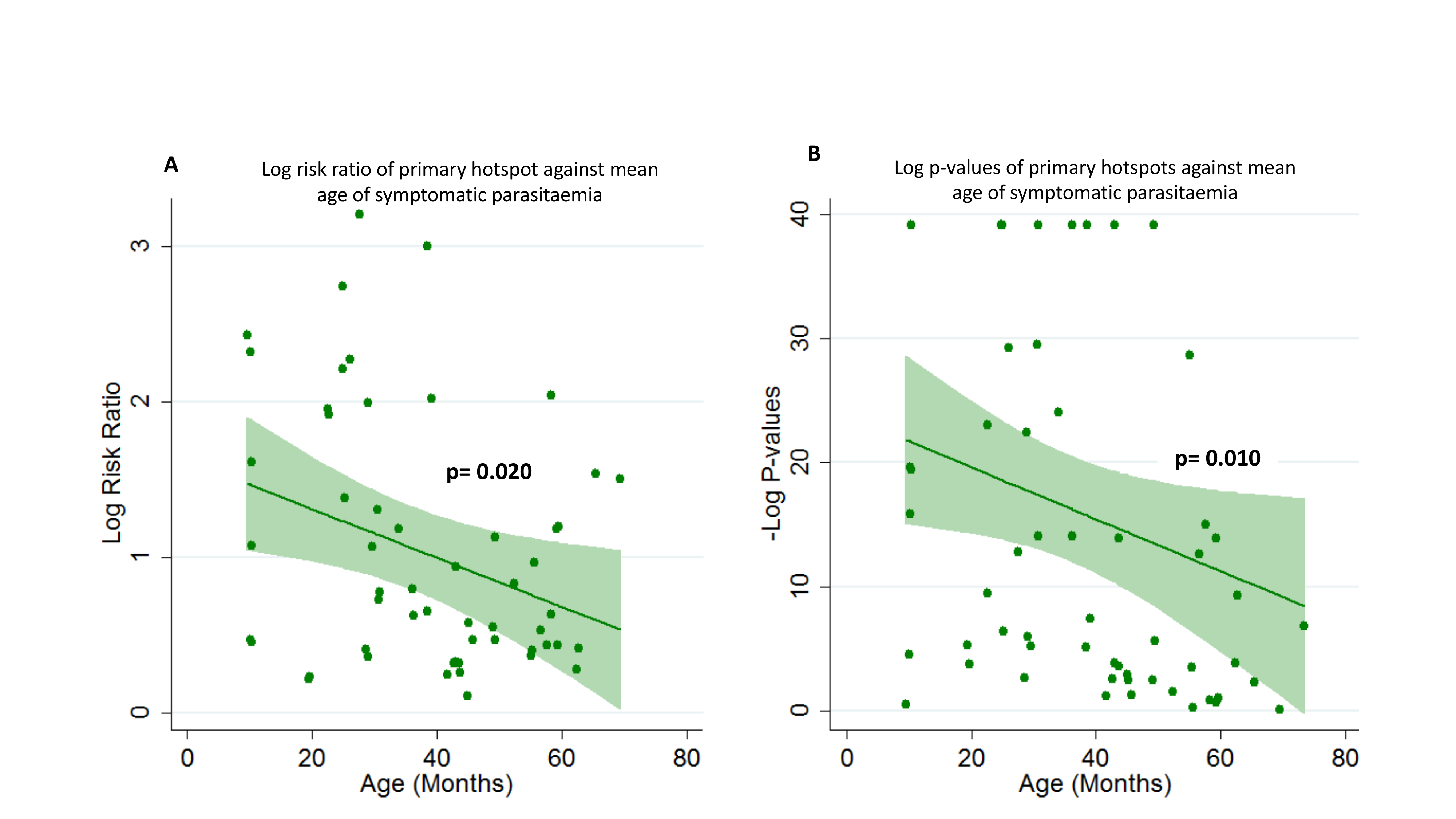

Supplement: Supplementary file 1 — Trends in parameters of primary hotspots over mean age of study participants. Panel A shows a scatter plot of log-transformed risk ratios against overall mean age. Panel B shows a scatter plot of log-transformed p values against overall mean age. The green line presents multiple fractional polynomial fits of age on malaria positive fraction (MPF) adjusted for the study design. Shaded areas in panels A and B represent 95% CIs. (TIFF 573 kb) [file 12916_2017_887_MOESM1_ESM.tiff]

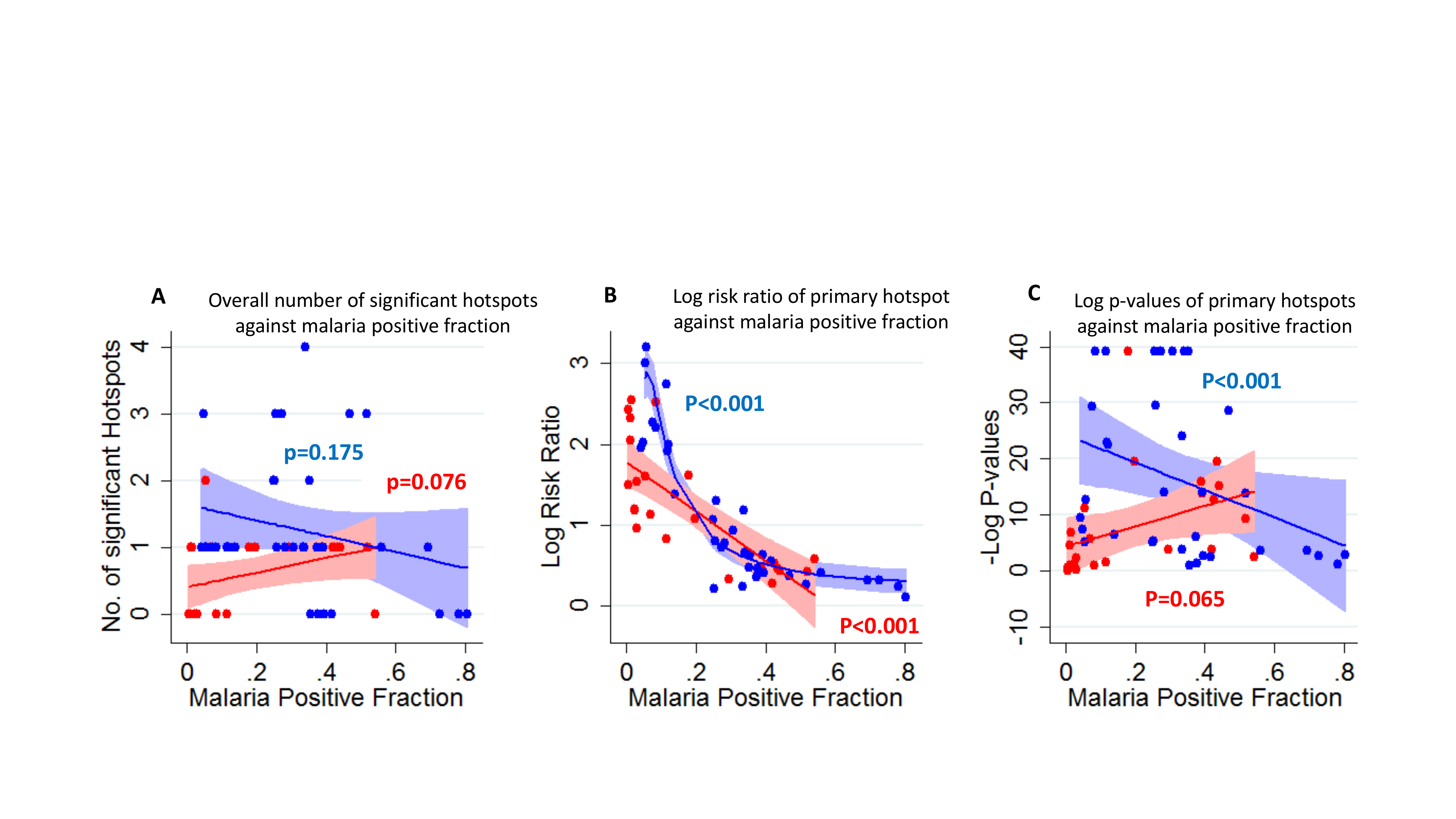

Supplement: Supplementary file 2 — Summary of malaria hotspots from symptomatic parasitaemia among passive (blue) and active (red) surveillance studies. Panel A shows a scatter plot of the number of significant hotspots against malaria positive fraction, panel B presents the log risk ratios of malaria within the primary hotspot against the malaria positive fraction and panel C presents the –log p values of the primary hotspots against malaria positive fraction. The blue and red lines in panels A, B and C show the fitted multiple fractional polynomial model predictions for passive and active case detection studies respectively. Shaded areas in panels A, B and C represent 95% CIs. (TIFF 821 kb) [file 12916_2017_887_MOESM2_ESM.tiff]

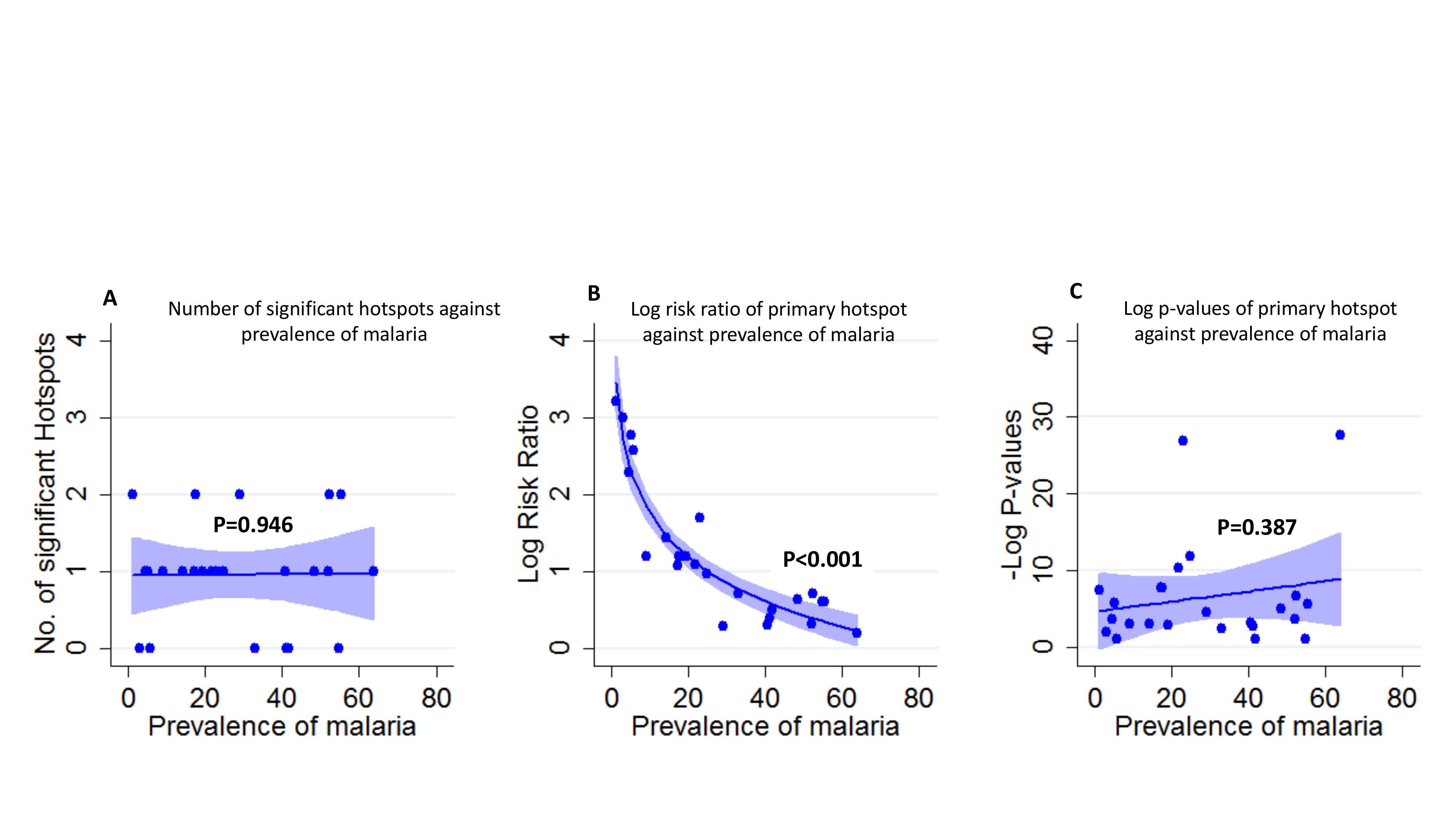

Supplement: Supplementary file 3 — Hotspots of asymptomatic parasitaemia. Panel A displays a scatter plot of the number of significant hotspots in each study dataset against parasite prevalence, panel B presents the log risk ratios of malaria within the primary hotspot against the parasite prevalence and panel C displays the –log (p values) of the primary hotspots against parasite prevalence. The blue lines in panels A, B and C show the fitted multiple fractional polynomial model predictions. Shaded areas in panels A, B and C represent 95% CIs. (TIFF 767 kb) [file 12916_2017_887_MOESM3_ESM.tiff]

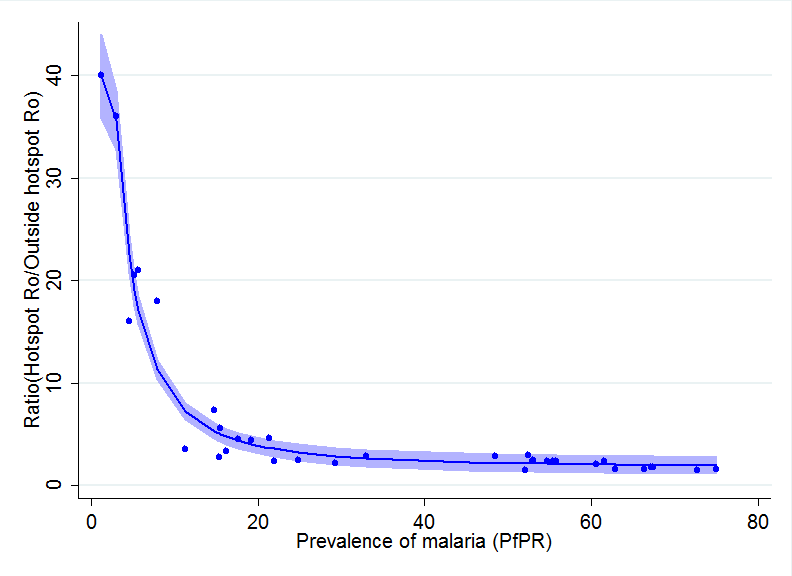

Supplement: Supplementary file 5 — Scatter plot of the ratio of log-transformed R 0 inside to outside the hotspot plotted against parasite prevalence. The blue line shows the fitted multiple fractional polynomial model predictions, and the shaded area represents 95% CIs. (TIF 1338 kb) [file 12916_2017_887_MOESM5_ESM.tif]

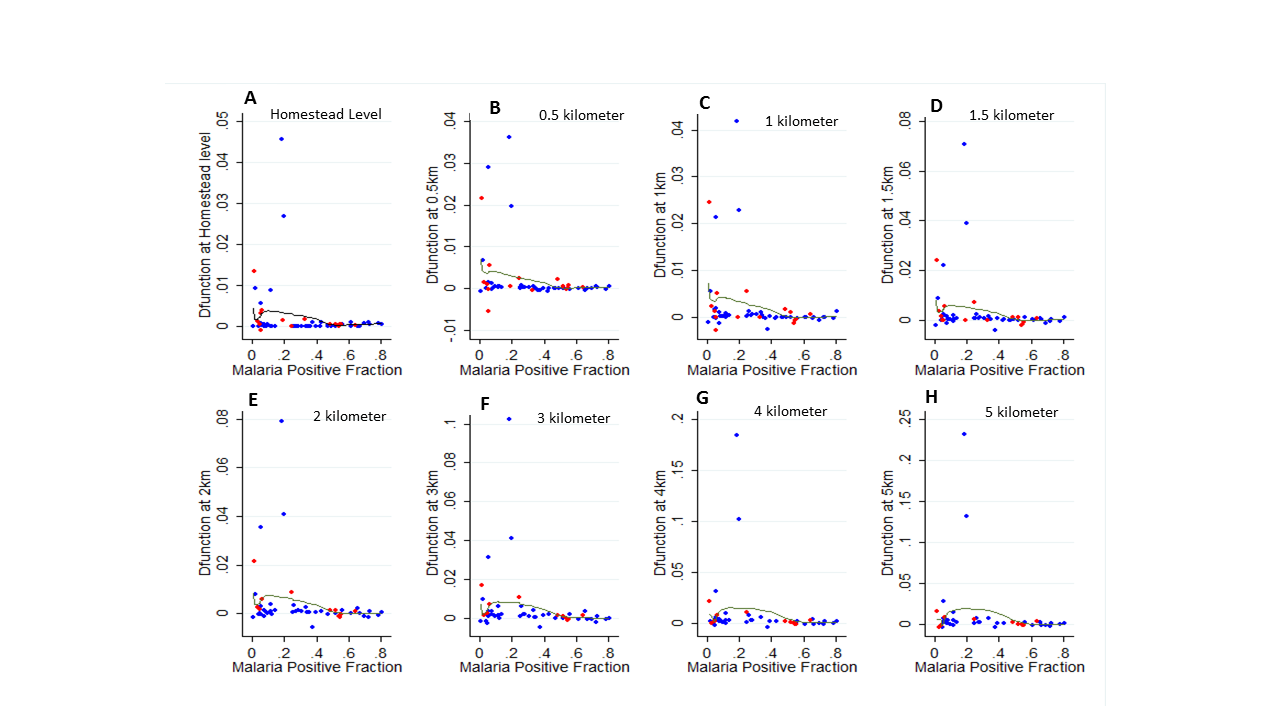

Supplement: Supplementary file 6 — Difference in K functions for cases and controls (D function) against malaria positive fraction. Panels A, B, C, D, E, F, G and H show the D function at homestead level, 0.5, 1, 1.5, 2, 3, 4 and 5 km distances for each dataset. The blue dots represent symptomatic parasitaemia datasets, while red dots represent asymptomatic parasitaemia datasets. (TIF 216 kb) [file 12916_2017_887_MOESM6_ESM.tif]

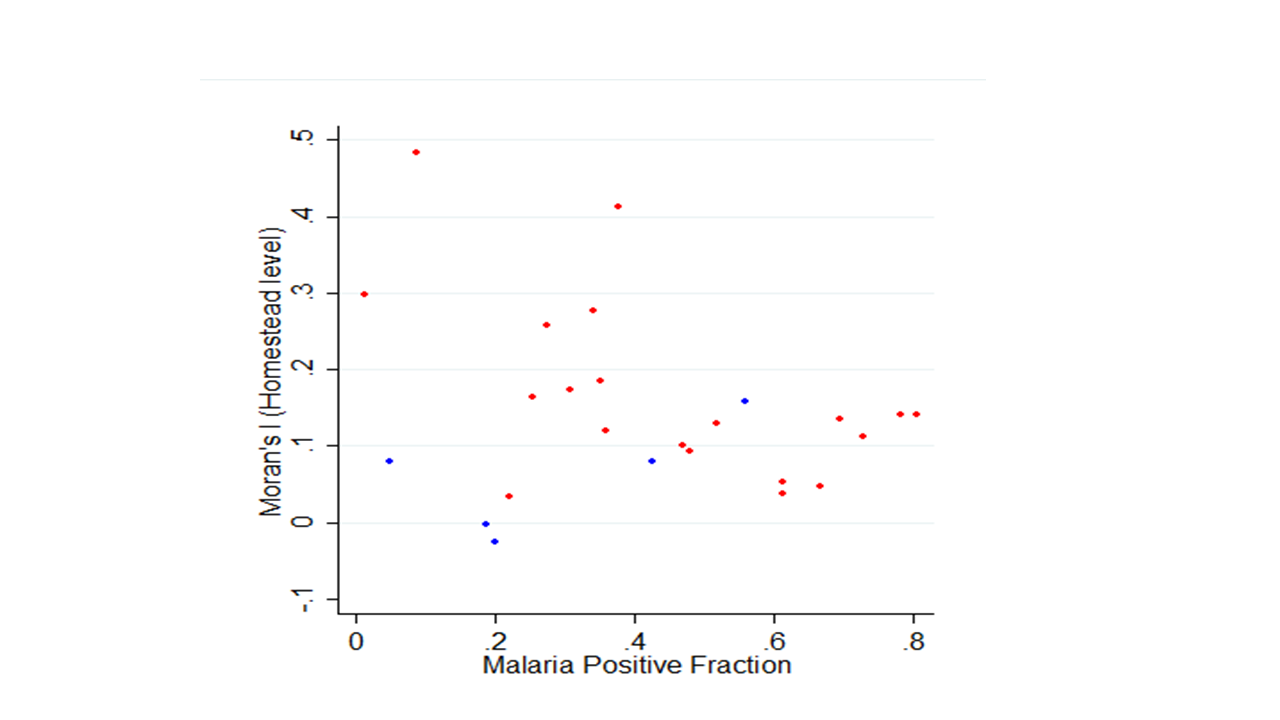

Supplement: Supplementary file 7 — Homestead level spatial autocorrelation of age in months for symptomatic individuals for the various studies. Red dots show significant autocorrelation, while blue dots show non-significant spatial autocorrelation. (TIF 90 kb) [file 12916_2017_887_MOESM7_ESM.tif]
