# Supplementary material for: Effect of transmission intensity on hotspots and micro-epidemiology of malaria in sub-Saharan Africa
Source: BMC Med. 2017 Jun 30;15:121. doi: 10.1186/s12916-017-0887-4 (PMC5492887; doi:10.1186/s12916-017-0887-4)
Supplement: Supplementary file 4 — Comparison between linear and the multiple fractional polynomial model fit. The p value shown derives from the log likelihood ratio test for a nested model with a fractional polynomial over the linear fit. (DOCX 12 kb) [file 12916_2017_887_MOESM4_ESM.docx]

**Additional File 4: Table S1:** Comparison between linear and the multiple fractional polynomial model fit. The P-value shown derives from the log-likelihood ratio test for a nested model with a fractional polynomial over the linear fit.

| Hotspots Parameter | Figure | Best fit fractional polynomial transformations | Deviance difference | P-value | Adjusted R^2^ |
| --- | --- | --- | --- | --- | --- |
| Number of hotspots | Fig 2A | MPF__1 = MPF^3 - 0.02058 | 9.181 | 0.04 | 0.1589 |
| Log risk Ratio | Fig 2B | MPF__1 = MPF - 0.27406  MPF__2 = MPF^2 - 0.07511 | 17.486 | 0.001 | 0.7382 |
| - log P-value | Fig 2C | MPF__1 =MPF^0.5 - 0.5235  MPF__2 = MPF- 0.27406 | 11.667 | 0.015 | 0.2792 |

*MPF refers to Malaria Positive Fraction and MPF__1 and MPF__2 are the fractional polynomial transformations for MPF.
